# Estimating the Spatial and Temporal Distribution of Species Richness within Sequoia and Kings Canyon National Parks

**DOI:** 10.1371/journal.pone.0112465

**Published:** 2014-12-03

**Authors:** Steve Wathen, James H. Thorne, Andrew Holguin, Mark W. Schwartz

**Affiliations:** 1 John Muir Institute of the Environment, University of California Davis, Davis, California, United States of America; 2 Information Center for the Environment, University of California Davis, Davis, California, United States of America; 3 Department of Environmental Science & Policy, University of California Davis, Davis, California, United States of America; Shanxi University, China

## Abstract

Evidence for significant losses of species richness or biodiversity, even within protected natural areas, is mounting. Managers are increasingly being asked to monitor biodiversity, yet estimating biodiversity is often prohibitively expensive. As a cost-effective option, we estimated the spatial and temporal distribution of species richness for four taxonomic groups (birds, mammals, herpetofauna (reptiles and amphibians), and plants) within Sequoia and Kings Canyon National Parks using only existing biological studies undertaken within the Parks and the Parks' long-term wildlife observation database. We used a rarefaction approach to model species richness for the four taxonomic groups and analyzed those groups by habitat type, elevation zone, and time period. We then mapped the spatial distributions of species richness values for the four taxonomic groups, as well as total species richness, for the Parks. We also estimated changes in species richness for birds, mammals, and herpetofauna since 1980. The modeled patterns of species richness either peaked at mid elevations (mammals, plants, and total species richness) or declined consistently with increasing elevation (herpetofauna and birds). Plants reached maximum species richness values at much higher elevations than did vertebrate taxa, and non-flying mammals reached maximum species richness values at higher elevations than did birds. Alpine plant communities, including sagebrush, had higher species richness values than did subalpine plant communities located below them in elevation. These results are supported by other papers published in the scientific literature. Perhaps reflecting climate change: birds and herpetofauna displayed declines in species richness since 1980 at low and middle elevations and mammals displayed declines in species richness since 1980 at all elevations.

## Introduction

Because there is evidence for decreasing biodiversity even within protected natural areas, changes in the patterns of species richness are increasingly seen as important to the management of natural areas [1]. Indeed, national parks in the United States are under a mandate to develop Natural Resource Condition Assessment (NRCA) reports that describe (1) current conditions for important park natural resources, including species richness or biodiversity; (2) the factors that influence the condition of those resources; (3) critical gaps in knowledge; and (4) the resources most in need of management attention.

These NRCA reports are meant to rely primarily upon existing data and expert judgment. This study resulted from a component of one such NRCA report for Sequoia and Kings Canyon National Parks (SEKI) [2]. The results presented here represent our estimates of past and current species richness within the Parks using existing plant and wildlife databases [3].

Our assessment used three preexisting data sets: (1) park-wide systematic surveys of particular taxonomic groups (e.g. plants, birds, etc.), (2) localized targeted studies of particular taxa, and (3) a large park wildlife observation database (WOD) containing over 66,500 records of chance wildlife or plant observations.

We used these three data sets to describe the current spatial distribution of species richness within the parks as well as temporal changes in the species richness of terrestrial vertebrates since 1980. To accomplish this, data was first divided up into one of four taxonomic bins (blocks): birds, mammals, herpetofauna (reptiles and amphibians), and vascular plants. Each taxonomic bin was then studied separately to investigate patterns based on (1) habitat type, (2) elevation zone, and (3) time period.

Since the various blocks of data described above were not sampled at the same levels of intensity (e.g. more records from one habitat type, elevation zone, or time period than another) we used rarefaction to arrive at estimates of species richness that were not biased based upon sampling intensity. Rarefaction is a randomized data resampling strategy used when comparing data with different numbers of observations (sample size) [4]–[7]. Rarefaction curves are plots of the number of species observed (y axis) as a function of the total number of samples collected (x axis). Rarefaction curves generally increase rapidly at first, as the most common species are encountered quickly. Rarefaction curves then curve toward an asymptote as much larger sample sizes are required before encountering the rarest taxa. Rarefaction assumes (1) that the number of occurrences of a species or larger taxon reflects sampling intensity, (2) that enough observations have been made overall, and (3) that all types of environments have been sampled adequately.

We compare our results with results derived from other studies of species richness in the Sierra Nevada Range. We then discuss what environmental drivers might be causing the species richness spatial patterns we and others have observed. Finally, we discuss the applicability of using this methodology for estimating species richness in other natural areas outside of national parks.

Although we analyzed species diversity (Simpsons index) as well as species richness, the resulting patterns were on the whole similar: (0.78<r^2^<0.95) [3]. Therefore we only discuss the results for species richness in this paper. Since the term “biodiversity” is generally used to describe species richness, rather than species diversity, we use “species richness” and “biodiversity” interchangeably.

We treat this assessment of species richness based on wildlife observation databases and biological studies as an exemplary case study because SEKI invested heavily in creating a large wildlife observation database. Therefore, examining the observations recorded by park rangers, scientists, and visitors for over a century provided an opportunity to examine the strengths and weaknesses of using non-systematic data to assess biodiversity. As more casual observation data sources emerge through the development of web-based citizen science databases (e.g. the National Phenological Network) there will be increasing opportunities to estimate species richness patterns using non-survey-based data.

## Materials and Methods

### Study site

**Figure 1:**
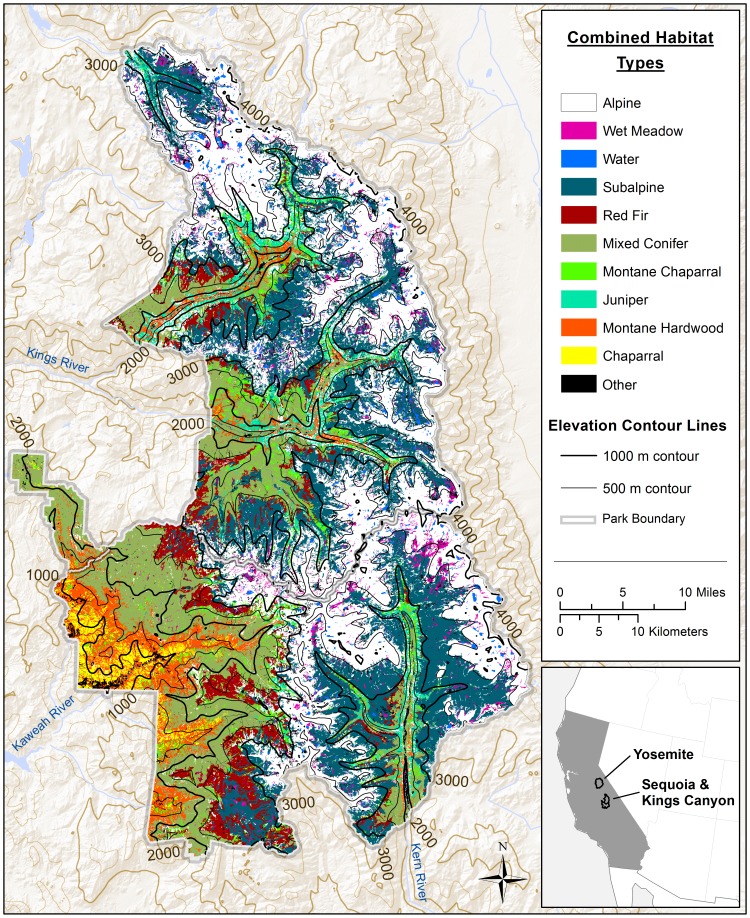
The spatial distribution of combined California wildlife habitat types within Sequoia Kings Canyon National Parks. The 26 California wildlife habitat types found within Sequoia Kings Canyon National Parks were merged into 11 “combined” habitat types to make elevation and vegetation patterns more comprehensible to the reader.

Sequoia and Kings Canyon National Parks cover 3504 km^2^ and are located adjacent to each other in the southern portion of California's Sierra Nevada Mountain Range. Kings Canyon National Park is located north of Sequoia National Park (Figure 1). Elevations within SEKI range from just above 400 m up to 4,418 m in elevation. The elevation of the highest point in SEKI, Mount Whitney, is also the highest point in the lower 48 states of the United States. Because the southern Sierra Nevada Range tilts upward to the east, most low elevation sites and roads within SEKI are found on the west side of the Parks, while Mount Whitney and most high elevation sites are located on the east side of the Parks.

Most of SEKI is rugged, rocky country, with shallow soils, and located at high elevations. Most of SEKI is covered by mixed conifer forests, subalpine forests, open alpine vegetation, or exposed rock. Indeed, more than half of SEKI is covered by alpine vegetation or exposed rock. Granitic rock outcrops occupy 85% of SEKI. The rest of the SEKI is underlain by metamorphic rock or glacial alluvium. Vegetation is usually sparse at the highest elevations.

Sequoia and Kings Canyon National Parks experience a Mediterranean climate characterized by long summer droughts, with moisture coming primarily as snow in the winter. Most of the visitation within SEKI is along roads at lower elevations located along the west side of the Parks. Access to most of the rest of SEKI is by steep trails. Most of the high country within SEKI is inaccessible in winter due to deep snow.

### Data sources by taxonomic group

We allocated records from the three data sets (park-wide systematic surveys, localized targeted studies of particular taxa, and chance wildlife observations) into one of four taxonomic groups: (1) birds, (2) mammals, (3) reptiles and amphibians (collectively known as herpetofauna), and (4) plants. Although low elevation sites on the west side of the Parks, near popular visitor destinations, contributed the largest number of data points, a good deal of data came from throughout the Parks, most being contributed by backcountry rangers.

The single largest source of data for wildlife species in SEKI was the Wildlife Observation Database (WOD). The WOD is composed of chance observations of wildlife, 90% of which were observed by National Park Service staff (Dave Graber, personal conversation on May 2, 2014). The WOD includes records dating as far back as the late 19th century through November 24, 2009. However, 96% of the records were collected since 1972. The WOD data accounted for 82% of the wildlife records used in this paper (Table 1) and the WOD was screened for accuracy by taxonomic specialists working for SEKI.

**Table 1:** Data sets and sample sizes used to estimate species richness values for birds, mammals, amphibians, reptiles and plants of Sequoia and Kings Canyon National Parks.

Category	Data Type	Dataset	Plots	Observations
**Animals**				
	**Single Species Observations**	SEKI Wildlife Observations Database	**-**	66,554
		Knapp Lakes Survey	**-**	12,378
		IBP Landbird Inventory	**-**	14,123
		**Total Animals**	**-**	**93,055**
**Plants**				
	**Vegetation Plots**	Stephenson Gradient Analysis	228	4,194
		Natural Resource Inventory (1985–1996)	627	15,210
		Vegetation Mapping (2000–2003)	423	9,088
		Wetland Ecological Integrity (2008–2009)	110	2,724
		Paired Meadow Plots (1985–2009)	10	3,102
	**Rapid Assessment/Special Purpose Plots**	Vankat/Roy Vegetation TransectsPlots (1969/1996)	76	425
		Vegetation Mapping (2001–2002)	123	721
		ibid Accuracy Assessment (2002–2004)	2,821	17,410
		Fire Effects Plots (1986–2009)	132	3,493
		Blister Rust Plots (1995–1999)	154	7,111
		Mineral King Analysis (1996–2000)	209	3,976
	**Single Species Observations**	SEKI Herbarium Holdings	-	3,689
		Norris and Brennan Special Status Plant Surveys	-	282
		Inventory and Monitoring Special Status Plant Surveys	-	94
		**Total Plants**	**4,913**	**71,519**
		**Total Animals and Plants**	**4,913**	**164,574**

Birds made up the most comprehensive data set of the three vertebrate groups examined in this study because birds are more visible than are many nocturnal mammals (e.g. bats), underground mammals (e.g. mice, voles, etc.), or quick moving and elusive amphibians and reptiles. In addition, many people are interested and knowledgeable about birds. Birds accounted for 67% of all WOD records (45,363 bird records covering 216 bird species). Another large source of data on birds came from a two-year systematic Parks-wide survey of birds conducted by bird experts from the Institute for Bird Populations during the springs of 2003 and 2004 (14,123 records covering 109 bird species) [8].

Mammals accounted for 22% of all records within the WOD. However, 50% of mammal records within the WOD were of six relatively easily observed and easily identified species: black bears (*Ursus americanus Pallas, 1780*), mule deer (*Odocoileus hemionus* Rafinesque, 1817), coyotes (*Canis latrans* Say, 1823), yellow-bellied marmots (*Marmota flaviventris* Audubon & Bachman, 1841), pikas (*Ochotona princeps* Richardson, 1828), and chickarees (*Tamiasciurus douglasii* Bachman, 1839). Small, nocturnal, and otherwise hard to observe mammals had, by contrast, relatively few records. As a consequence, most (72%) mammal species within the WOD are represented by ≤20 records.

Amphibians and reptiles were the most poorly represented of the terrestrial vertebrate groups, accounting for only 6% of WOD records (n = 3708). A focused study of amphibians and reptiles around high elevation lakes within SEKI was not included in from our analyses because including so many records from one elevation zone would have created undue elevational bias.

The southern Sierra Nevada Range has particularly high levels of plant diversity. The southern Sierra Nevada Range has between 250–330 endemic species [9]. There are four times as many plant species as vertebrate species living in SEKI and plants had almost as many records (75%) as did all the terrestrial vertebrates combined (71,519 versus 93,055 records, respectively). We identified three sources of plant data: (1) herbarium specimens collected within SEKI, (2) records from localized targeted studies of plants, and (3) park-wide systematic surveys undertaken for various purposes (vegetation mapping, fire effects monitoring plots, etc.). However, since most of the plant records were observed after 1985, this did not allow for a study of changes in plant species richness since the 1980s, as was possible for terrestrial vertebrates.

We created a vegetation map for SEKI by combining geospatial data from both the Parks (“sekigeodata.zip” folder: *Vegetation Characterization Products for the Sequoia and Kings Canyon National Parks*, https://irma.nps.gov/App/Reference/Profile/2168871, visited May 6, 2014) and the California Wildlife Habitat Relationships System (*Wildlife Habitats: California Wildlife Habitat Relationships System*, http://www.dfg.ca.gov/biogeodata/cwhr/wildlife_habitats.asp, visited May 6, 2014).

Our vegetation map originally consisted of 26 habitat types (Table 2). For visual simplicity only (we analyzed all 26 habitat types), we consolidated the 26 habitat types into eleven “combined” habitat types, with eight of the rarest of the original 26 habitat types pooled together into the “other” combined habitat type (Table 2, Figure 1).

**Table 2:** Species richness values and (standard errors) for each of the four taxonomic groups by California wildlife habitat type.

Habitat Type	Plant	Bird	Mammal	Herptofauna	Combined Habitat
Alpine (Barren)	154.7 (6.4)	84.7 (4.1)	28.2 (2.5)	13.8 (1.5)	Alpine
Sagebrush	157.3 (5.9)	77.5 (2.9)	NE	NE	Alpine
Wet Meadow	145.9 (6.2)	89.4 (3.6)	27.2 (2.0)	9.7 (1.6)	Wet Meadow
Water	194.1 (4.7)	100.9(4.0)	32.5(2.4)	8.2(1.4)	Water
Subalpine Forest	117.8 (6.1)	70.1 (3.7)	23.8 (2.2)	8.2 (1.4)	Subalpine
Lodgepole	132.5 (6.3)	75.6 (3.6)	29.6 (2.4)	13.6 (1.5)	Subalpine
Aspen	115.2 (3.8)	NE	NE	NE	Subalpine
Red Fir	115.1 (6.1)	80.0 (3.7)	28.8 (2.0)	NE	Red Fir
Sierra Mixed Conifer	130.8 (6.4)	86.3 (3.8)	33.6 (2.6)	20.2 (1.4)	Mixed Conifer
Giant Sequoia	101.6 (5.4)	73.8 (3.5)	33.0 (2.5)	18.4 (0.7)	Mixed Conifer
White Fir	129.7(5.7)	77.7 (3.5)	27.9 (0.3)	NE	Mixed Conifer
Jeffrey Pine	122.5 (6.3)	82.6 (3.7)	28.9 (2.2)	17.0 (1.3)	Mixed Conifer
Juniper	112.5 (5.6)	74.3 (0.8)	NE	NE	Juniper
Pinyon - Juniper	106.0 (0.0)	NE	NE	NE	Juniper
Montane Hardwood	145.4 (6.5)	106 (3.9)	28.3 (2.6)	20.4 (1.7)	Montane Hardwood
Montane Chaparral	128.6 (6.1)	90.6 (3.6)	28.7 (1.3)	18.9 (0.3)	Montane Chaparral
Mixed Chaparral	111.6 (5.2)	98.8 (2.0)	NE	NE	Chaparral
Chamise Chaparral	119.8 (5.0)	87.1 (3.2)	19.9 (1.6)	18.9 (0.9)	Chaparral
Alpine Dwarf Shrub	152.8 (3.2)	NE	NE	NE	Other
Montane Riparian	176.5 (6.3)	94.2 (3.7)	38.3 (2.3)	19.4 (0.4)	Other
Ponderosa Pine	NE	72.9 (2.2)	NE	NE	Other
Valley Foothill Riparian	NE	NE	NE	NE	Other
Perennial Grass	157.8 (4.9)	92.4 (3.0)	NE	NE	Other
Blue Oak Woodland	128.6 (4.7)	84.4 (3.9)	20.2 (1.9)	18.9 (1.6)	Other
Annual Grassland	NE	NE	NE	NE	Other
Urban	NE	76.8 (3.4)	NE	NE	Other

“NE” = Not Estimated = habitat types with insufficient sample sizes to estimate species richness. “Combined Habitat” = similar habitat types located close to each that were combined together for ease of visualization in Figure 1. “Other” = habitat types that made up relatively little of the Parks and so were combined together.

We refer to the California Wildlife Habitat type “barrens” as “alpine” habitat type in this paper because almost all of the “barren” area within SEKI was of rock outcrops located within the alpine zone. There were rock cliffs at middle elevations and disturbed sites at low elevations that were also designated as barren within SEKI but they constituted little of the area within the Parks and thus were not mapped separately.

More detailed vegetation classification schemes for SEKI, with many more vegetation types, were available. However, there were not enough records per vegetation type to make confident estimates of species richness at those finer scales (*sekivegmap, 2010*, http://www1.usgs.gov/vip/seki/sekivegmap.pdf, visited May 6, 2014).

The data used in our analyses is owned and maintained by the National Park Service and is freely available by contacting:

Chief of Science and Natural Resources Management

Sequoia and Kings Canyon National Parks

47050 Generals Highway

Three Rivers, CA 93271-9700

559-565-3341

### Data analysis

To assess the distribution of species richness within SEKI, we divided data by taxonomic groups and then analyzed them separately by (1) habitat type, (2) elevation zone, and (3) time periods. Vegetation was divided into the 26 wildlife habitat types referred to above (eventually reduced to 11 combined habitat types for visualization purposes in Figure 1). Elevations were divided into 500 m elevation zones. Four time periods were distinguished: (1) for all observations recorded before 1980 and for the decades of the (2) 1980s, (3) 1990s, (4) 2000s,respectively.

Since data described above were not equally sampled (e.g. more records from one habitat type, elevation zone, or time period than another), we used rarefaction to arrive at estimates of species richness without biases due to differences in sampling intensity.

**Figure 2:**
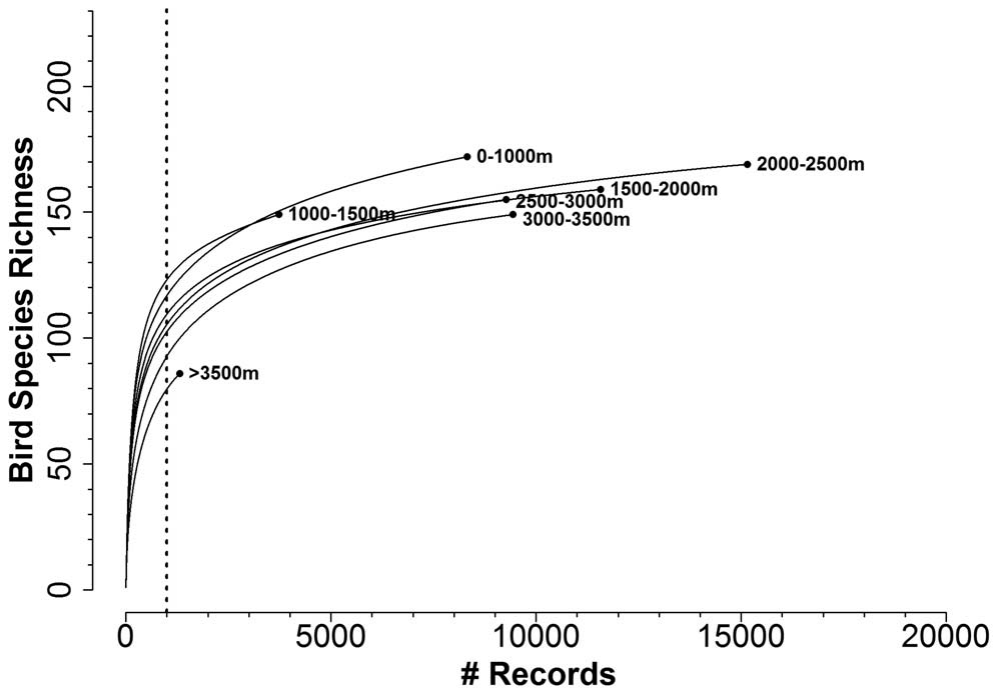
Example of rarefaction curves used in the analyses: Rarefaction of bird diversity by elevation. Each line represents all the records from within a given 500 m elevation band. Each curve estimates the number of species that would be encountered from a given number of bird recorded observations. The length of each line represents the total number of bird records for that elevation zone. The vertical dotted line represents the minimum threshold sample sizes used to estimate species richness so as to include all curves.

To estimate species richness values using rarefaction, one must choose a minimum sampling size, i.e. a minimum number of records that all members of a given bin must meet. These minimum threshold sample sizes were different for each analysis (Table 3). We selected minimum sampling thresholds based upon the shape of the rarefaction curves with the goal of estimating species richness for as much the area within SEKI as possible for each analysis (Figure 2).

**Table 3:** Rarefaction threshold cutoff values sample sizes for habitat types, elevation zones, and time periods for plants, birds, mammals, and herptofauna.

Analysis by	Plants	Birds	Mammals	Herptofauna
Habitat Types	275	400	250	100
Elevation Zones	2000	1000	500	250
Time Periods	NE	400	150	50
Number of the 26 Wildlife Habitat Types Analyzed	22	21	15	13

Species richness values from data bins with sample sizes below these threshold values were not stable and so were not estimated. Thus, plants had four (26 possible-22 estimated) California wildlife habitat types with insufficient data to estimate species richness.

Rarefaction analysis was done in R using the ‘rarefy’ function in the ‘vegan’ package [10]. This function is based on the rarefaction formula developed by Oksanen and Blanchet et al. [11] and the standard errors developed by Hurlbert [12]. In the end, we were able to estimate species richness values for 85%–97% of SEKI's land area depending upon the taxonomic group. The herpetofauna, with the fewest records and lowest number of species at high elevations, also had the lowest park coverage.

**Figure 3:**
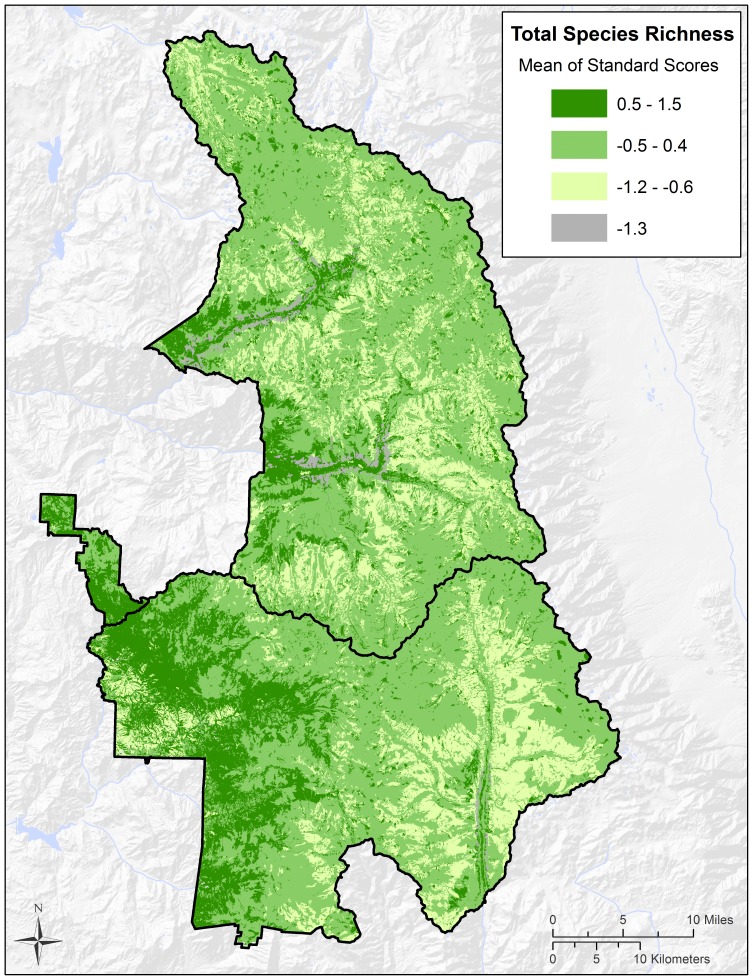
The distribution of total species richness by habitat type within Sequoia and Kings Canyon National Parks. Total species richness values (indices) represent the average of standardized species richness values for all four taxonomic groups combined: birds, mammals, herpetofauna and plants.

We also estimated total species richness values for the Parks by combining species richness values for all four taxonomic groups. To accomplish this we standardized values using standard scores for each of the 26 habitat types by taxonomic group. We then averaged the values for the four taxonomic groups to arrive at a standardized total species richness value (index) for each habitat type. Finally we ranked these total species richness values into four classes and mapped them accordingly (Figure 3).

In addition, we analyzed how much individual species presence turned over between adjacent elevation zones. We also analyzed how much species richness changed from January, 1980 to November, 2009 for terrestrial vertebrates.

The geospatial coverages used in our analyses belong to the National Park Service and can be found online at http://www.usgs.gov/core_science_systems/csas/vip/parks/seki.html. Our methods for initially analyzing the data can be found in Appendix 20B of “A Natural Resource Condition Assessment for Sequoia and Kings Canyon National Parks,” which can be downloaded at irmafiles.nps.gov/reference/holding/474918. Subsequent analyses can be obtained by contacting the corresponding author of this paper.

## Results

**Figure 4:**
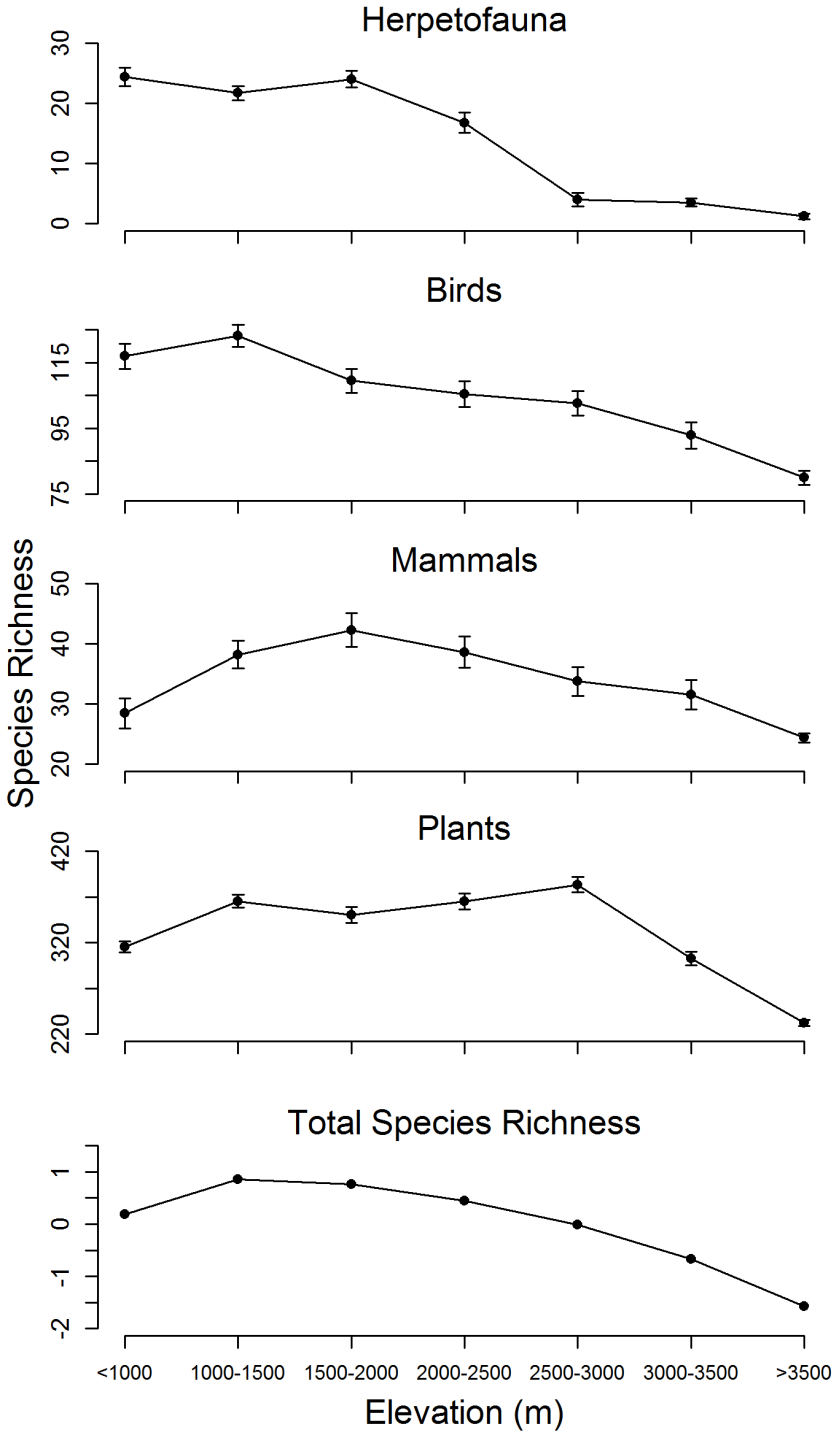
Estimated species richness values by elevation zone. Species richness values estimated using rarefaction for amphibians and reptiles (herpetofauna), mammals, birds, plants, and total species richness.

**Figure 5:**
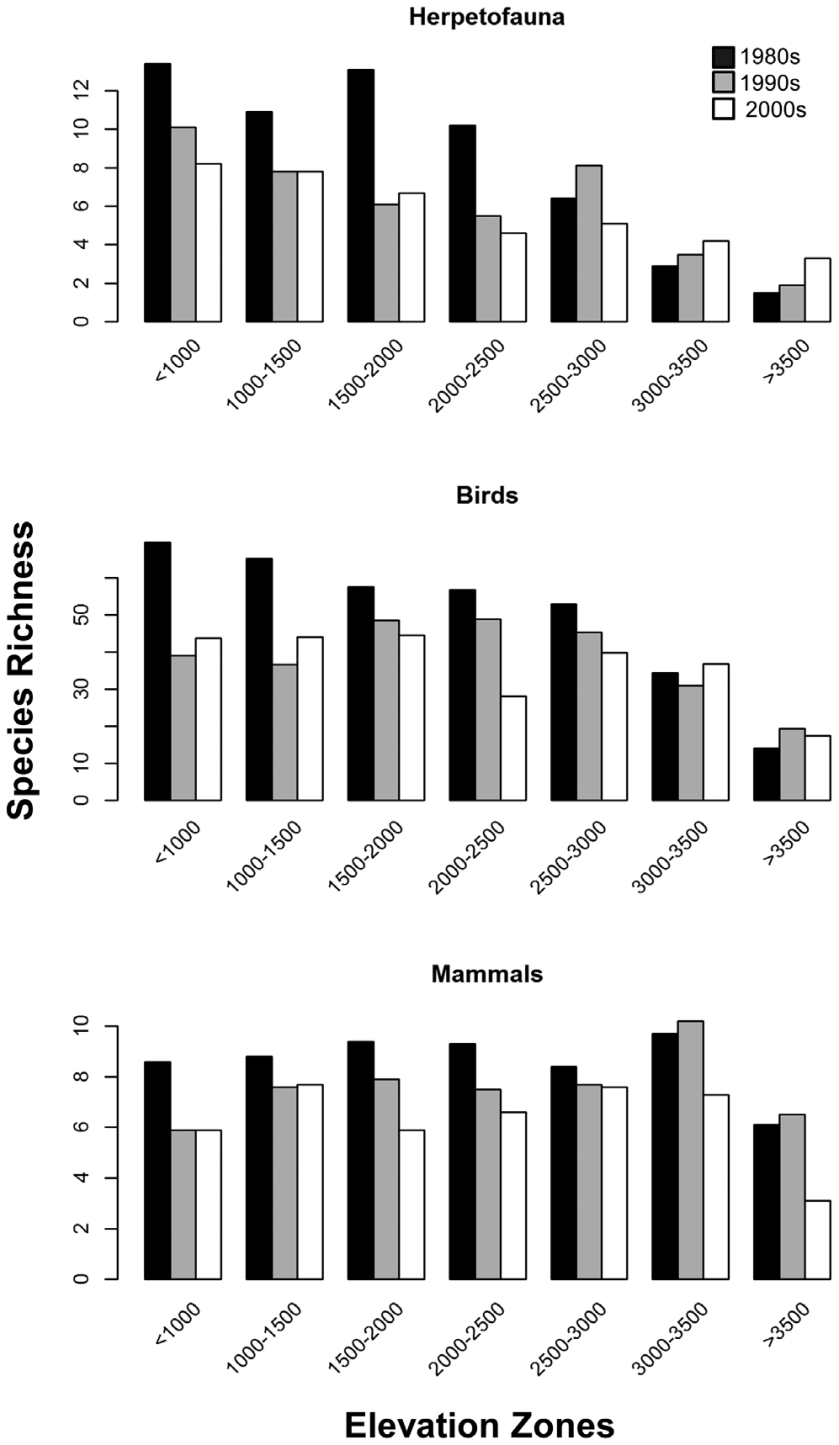
Estimated changes in species richness of birds, mammals, and herpetofauna through time. Species richness values were estimated for each 500 m elevation zone.

The dominant patterns we observed were that birds, amphibians, and reptiles had their highest species richness values at low to middle elevations, while mammals and plants had their highest values at middle to high elevations, respectively (Figure 4). Our temporal analyses suggest a relatively simple pattern of decreasing species richness at low and medium elevations since 1980 for all three vertebrate groups (Figure 5). Mammals displayed decreasing species richness at high elevations as well. In general, there was less decrease in species richness at high elevations since 1980, and sometimes increases. There was insufficient data for a temporal analysis of plant species richness pre and post 1980.

### Correlations

As expected, our estimates of total species richness were positively correlated with the absolute number of records in the WOD for most vegetation habitat types (r^2^ = >0.96). We did not estimate species richness values for some habitat types because of few occurrences (i.e. <100 records within a habitat type). However, most of these habitat types accounted for very little of the area within SEKI. In the end we estimated species richness for 22 of 26 habitat types for plants, 21 of 26 habitat types for birds, 15 of 26 habitat types for mammals, and 10 of 26 habitat types for amphibians and reptiles (Table 2).

Sampling intensity was approximately equal across most habitat types for terrestrial vertebrates. There was a near linear relationship between the area occupied by a given habitat type within the Parks and the number of records of vertebrates for that habitat type, with the exception of alpine and subalpine habitat types. Alpine and subalpine habitat types, the two largest habitat types within the Parks (Figure 1), had relatively low numbers of records given their large areal extent. Though two-thirds of SEKI is located above 2,500 m in elevation, only one-third of vertebrate records in the WOD originated from above 2,500 m. Low sampling density is likely a combination of both low vertebrate densities as well as fewer humans to observe vertebrates above 2,500 m in elevation.

### Spatial distributions

Species richness values for birds, amphibians, and reptiles were high within riparian areas, located within major river canyons, at low and middle elevations. Bird and mammal species richness values peaked within the 1,000–1,500 m and 1,500–2,000 m elevation zones, respectively, and then declined consistently (monotonically) with increasing elevation. Amphibian and reptile species richness values were high up to ∼2,000 m in elevation and then also declined monotonically with increasing elevation (Figure 4).

The pattern of distribution of species richness for plants was different from those for birds, amphibians, reptiles, and mammals. Plant species richness increased to 1,000–1,500 m in elevation (chaparral and montane hardwood vegetation), stayed approximately the same between 1,500 m and 2,500 m in elevation (mixed conifer and montane chaparral vegetation), peaked within the 2,500–3,000 m elevation zone (mixed conifer, red fir, and subalpine vegetation), and then declined with increasing elevation (alpine vegetation)(Figures 1 and 4). Rare plant taxa were also found in greatest numbers within the 2,500–3,000 m elevation zone in conjunction with peak plant species richness.

Similar to the pattern of species richness noted for birds, above, total species richness (vertebrate groups plus plants) was highest at ∼1,000–1,500 m in elevation. Total species richness was also high within canyon riparian areas at middle elevations. Total species richness then declined monotonically with increasing elevation (Figures 3 and 4). On Figure 3, the dark green color at low elevations, on the southwestern side of the Parks, appears to represent montane hardwood habitat type. The same dark green color at the bottom of river canyons located further north on the west side of the Parks appears to represent montane hardwood, riparian, or mixed conifer habitat types. The gray color within canyons, located just above canyon bottoms, appears to represent Sierra juniper and/or pinyon-juniper habitat types located on dry toe slopes. The pale yellow color found higher above the canyons appears to represent subalpine vegetation. Alpine tundra appears to have higher total species richness values than the subalpine vegetation located just below it in elevation.

On the basis of habitat types present within SEKI, we found that the following habitat types had the highest total species richness values: montane hardwoods at low elevations; Jeffrey pine forests, montane chaparral, and Sierra mixed conifer forests at middle elevations; alpine tundra, and wet meadows at high elevations; and perennial grasslands and montane riparian habitats located at all elevations (Table 2, Figure 1).

### Turnover between elevation zones

**Figure 6:**
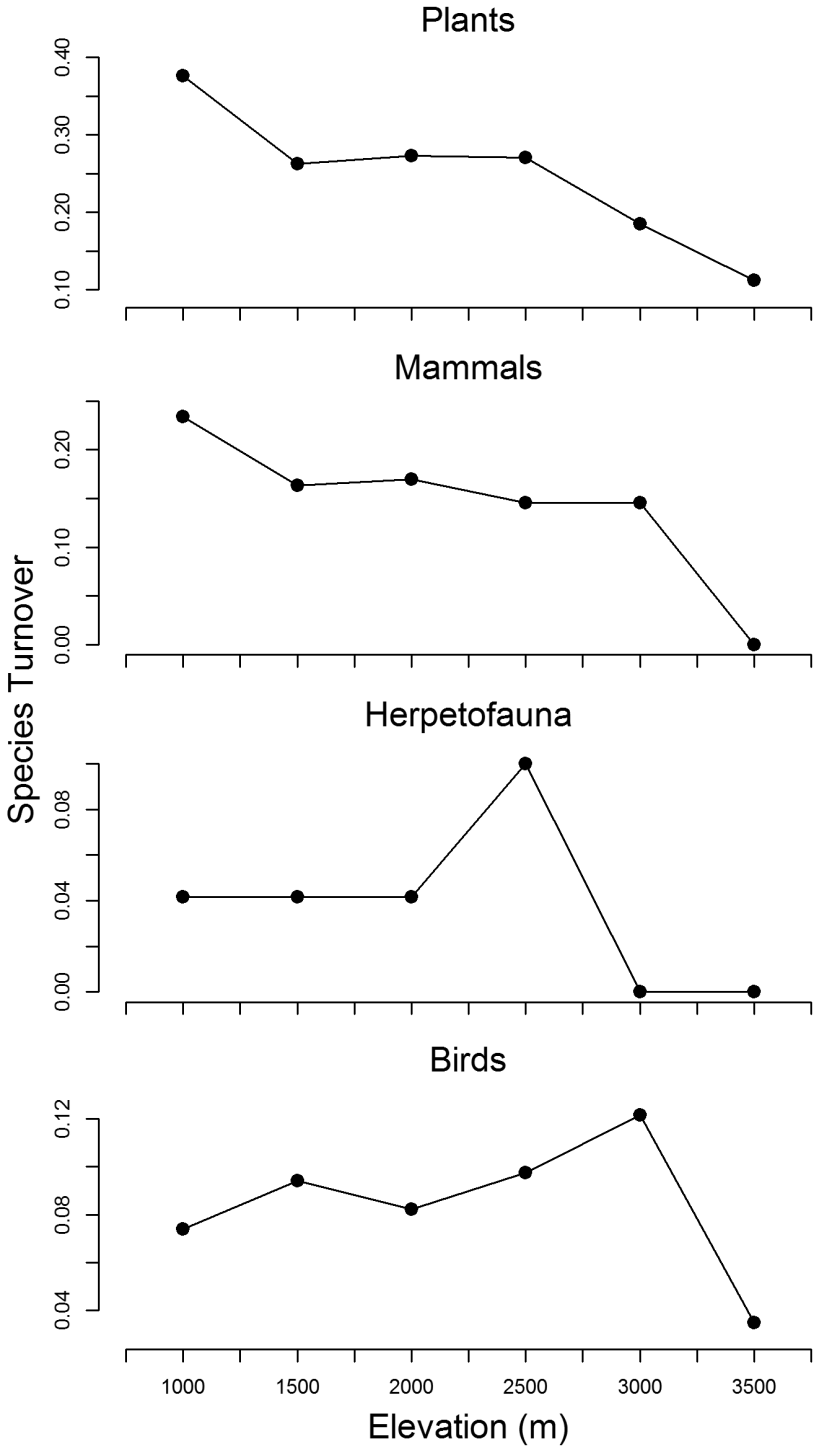
Estimated patterns of species turnover between elevation zones by taxonomic group. Values were calculated using the *Sørensen* dissimilarity *index*.

Our results suggest that patterns of species turnover between elevation zones differed substantially between taxonomic groups (Figure 6). Mammals and plants had their greatest turnover (dissimilarity) at 1,000 m in elevation, with turnover decreasing monotonically with increasing elevation. Conversely, bird, amphibians, and reptiles had low turnover rates at low elevations and their highest turnover rates at high elevations.

Turnover rates for amphibians and reptiles were highest, though never very high, at 2,500 m in elevation. There are few species of amphibians and reptiles sampled at the highest elevations, apparently due to cold temperature, resulting in little to no turnover of herpetofauna at 3,000 m or above in elevation.

Turnover rate for birds was highest at 3,000 m in elevation. The low turnover rate of birds at low elevations might be due to the relatively benign environmental conditions existing at low elevations, as well as the ability of birds to move rapidly and relatively safely around the landscape. Consequently, there would be a better chance for a given species of bird to be observed in multiple habitat types, than would be case for a species of some other taxonomic groups, all other things being equal (Figure 4).

### Temporal changes

Our results suggest that the number of bird species decreased from 1980 to the present at elevations below 3,000 m, while remaining stable or increasing above 3,000 m in elevation (Figure 5). This decrease in the number of bird species recorded at below 3,000 m has taken place despite an increase in the number of bird observations recorded since 1980. Increases in bird species richness above 3,000 m might reflect warming conditions.

Similarly, the number of amphibian and reptile species appears to have decreased from 1980 to the present at elevations below 2,500 m, while remaining stable or higher above 3,000 m. Again, this pattern might reflect warming conditions.

Mammal species richness has decreased since 1980 within all elevation zones. However, mammal species richness above 3,000 m appears to have increased in the 1990s, followed by sharp declines in the 2000s. Our analysis suggests that total vertebrate species richness values (birds, herpetofauna, and mammals) has decreased substantially below 3,000 m and remained ∼stable above 3,000 m in elevation.

## Discussion

### Spatial patterns of species richness worldwide and within SEKI

Species richness and species diversity values are highest at the equator and decrease poleward. Temperatures differences are thought to be one of the main causes for this trend, though researchers are still studying the drivers involved [13]. Since temperatures tend to decrease with elevation, it was initially assumed by many early ecologists that species richness should, similarly, decrease with increasing elevation [14].

In 1995, Rahbek [15] noticed that species richness and species diversity did not always follow a simple pattern of consistent decline (monotonic decrease) with increasing elevation. Rahbek reviewed 96 studies encompassing many taxa worldwide. After standardizing those studies for area and sampling effort, Rahbek found that most species richness curves were hump-shaped, i.e. unimodal. Rahbek also found that maximum species richness occurred at intermediate or lower elevations in half of the studies he reviewed. In 2005, Rahbek [16] revisited his earlier analysis and found that distributions where species richness decreased monotonically from low to high elevations was the next most common pattern of species richness change with elevation. Since Rahbek's initial paper [15], others have found similar results [17], [18].

Guo et al. [19] analyzed 443 elevational species richness distributions worldwide that involved a variety of taxa. They found that “positive” unimodal distributions, defined as where species richness peaked at some point below the mid-elevation of transects, occurred in 70% of the studies analyzed within the northern hemisphere (patterns were different for the southern hemisphere). Another 20% of northern hemisphere studies exhibited monotonically decreasing species richness values beginning at lowest elevations. The remaining 10% of studies exhibited a variety of other distributions.

Guo et al. also found that positive unimodal distributions were most common between 20° and 40° latitude in the northern hemisphere. The greater the elevational range surveyed and the larger the number of taxonomic groups sampled, the more unimodal distributions became. They also found that the lower in elevation that transects began, and to a lesser extent the higher in elevation that transects ended, the higher was the elevation at which maximum species richness occurred. The elevations of our study, and of SEKI, began at ∼500 m and ended at over 4,000 m.

Our findings agree with many of those reported by Rahbek and Guo et al. All five taxonomic distributions were either hump-shaped (mammals, plants, and total species richness values) or declined monotonically from low elevation (herpetofauna and bird values), which Rahbek reported occurred in 90% of cases. Herpetofauna, birds, mammals, and total species richness values peaked between the base elevation of the Parks and the mid-elevation level of sampling, which Guo et al. reported was the most common pattern (positive unimodal distributions) at our latitude in the northern hemisphere. Only plant species richness peaked above the mid-elevation level of sampling, which Guo et al. would have labeled a negative unimodal distribution. Our study analyzed many species over a large elevational range and had mostly unimodal distributions, agreeing with Guo et al as well.

Das et al. [20] found that forests of the Sierra Nevada Range are water limited at low elevations and temperature limited at high elevations. Given that our study area extends from low dry foothills to high cold mountains, species richness may be constrained within SEKI at both low and high elevations by precipitation and temperature, respectively.

Guo et al. also compared distribution patterns between different taxa. They found that species richness distributions did not vary significantly between plants and animals, nor between different plant life forms (trees, shrubs, etc.). Amongst vertebrates, however, non-flying mammals had a higher proportion of positive unimodal distributions (76%) than bats (50%) or birds (40%). Guo et al. also reported that the elevations at which species richness peaked were generally higher for vertebrates than for invertebrates, for plants than for animals, and for herbs (and to a lesser extent shrubs) than for trees.

Our findings generally agree with those of Guo et al. In our study, plants reached maximum species richness values at closer to the upper end of the elevation range sampled than did vertebrate animals. Alpine herbs and/or small shrubs had higher species richness values than did subalpine trees located just below them in elevation. However, mammals, composed of mostly non-flyers in our study, reached peak species richness values at a higher elevation than did birds.

Also, our pattern of bird species richness by elevation generally agreed with that reported in the literature for the southern Sierra Nevada. In 2003 and 2009, Tingley et al. [21]–[23] resurveyed avian sites located near and within SEKI that had been originally surveyed by Grinnell et al. in the early 1900s [24]. Tingley and Beissinger [22] found that bird species richness was highest at 994 m in elevation, i.e. at low elevations, which agrees with both our results (1,000–1,500 m) and what Grinnell et al. reported early in the 20th Century.

### Drivers of gradients in species richness and species turnover

Richerson & Lum [25] found that annual mean values of temperature and precipitation were the more important predictors of plant species richness in California. Rahbek [15] attributed the high frequency of hump-shaped distributions of species richness with elevation in part to the fact that maximum precipitation, a major driver of productivity, often occurs at some intermediate elevation on mountains. Sanchez-Gonzalez [26] concluded that the distribution of species richness on mountains is influenced most by the decreases in temperature resulting from increasing elevation, precipitation patterns, and soil type.

Other researchers have emphasized additional variables affecting the distribution of species richness. Both Rahbek [16] and Lomolino [27] found that species richness patterns often varied appreciably depending upon what taxonomic group or taxonomic subgroup was studied, various local site characteristics, and the scale (grain) of the study undertaken. Both Sanders and Rahbek [13] and Lomolino [27] concluded that evolutionary and site history can also affect the distribution of species richness.

Some researchers have concluded that resource abundance might be a factor in allowing more or fewer species to persist in an area [28]–[30]. Hamilton and Perrot [31] hypothesized that environmental conditions might be more favorable for life at low to middle elevations, thus allowing for a greater number of species to exist there, but that fewer species can persist under harsher conditions at higher elevations. This might help explain the dominance of hump-shaped species richness distributions.

Some researchers have argued that the turnover rates between sites might be an indication of how many species can exist together. High turnover rates between elevation zones might indicate that conditions are such that only a relatively few species can exist in any one elevation zone [27]. Others would argue that high turnover rates at low elevations might indicate greater niche diversity.

In our analysis, birds had their highest species richness at low elevations (1,000–1,500 m), while bird species turnover peaked at 3,000 m (Figures 4 and 6). The high turnover at high elevations may be because relatively few bird species are adapted to live at higher elevations or because resources are scarcer at high elevations. The number of bird species living above 3,000 m may have increased since 1980, perhaps in response to amelioration of harsh conditions at high elevations through warming (Figure 5).

Herptofauna were relatively species rich below 2,000 m but turnover was highest above 2,500 m. Both observations may be because relatively few amphibians and reptiles are adapted to living at colder high elevations and the species that exist there are adapted to particular niches. Again, the number of herpetofauna species may have increased since 1980 at high elevations, perhaps in response to global warming (Figure 5).

In contrast, mammal and plants exhibited their greatest turnover at low elevations (∼1,000 m) and rates decreased monotonically with elevation, perhaps reflecting greater niche diversity. Mammal and total species richness peaked at relatively low elevations, while plant diversity peaked at high elevation.

### Temporal changes

In 2003 and 2009, Tingley and Beissinger [22] resurveyed 77 bird sites in three areas of the Sierra Nevada near and within Lassen, Yosemite, Sequoia, and Kings Canyon National Parks that were previously surveyed in 1911 and 1916 by Grinnell et al. [24]. After accounting for changes in species detectability, Tingley and Beissinger found that bird species richness had declined at all elevations in the Sierra Nevada over the 20th century. Individual survey sites had lost from five to 15 bird species per site, with a median loss of 2.6 species. Species turnover on these sites during the same time period had been even greater, ranging from 20%–49% species turnover, with a median value of 35% species turnover. They found that these changes were greatest at both low elevations and high elevations. Austin and Boiano et al. [32] determined that 30 species of birds have been lost from SEKI and 12 non-native species of birds introduced.

Our results suggest that the number of bird species in SEKI decreased from 1980 to the present at elevations below 3,000 m (especially at low elevations) but not at elevations above 3,000 m, where bird species richness may have increased (Figure 5). This decrease in the number of bird species recorded at low and middle elevations has occurred despite an increase in the number of recorded observations of birds within the WOD since 1980. Stability or slight increases in bird species richness above 3,000 m could reflect warming at high elevations as a result of human-caused climate change, warmer conditions allowing more bird species to exist there. The California condor (*Gymnogyps californianus* Shaw, 1797) had 42 recorded observations prior to 1982 but was later extirpated from the Parks (Grinnell described the decline of the California condor in 1912 [33]). However, individual condors, reintroduced elsewhere, are occasionally observed flying over the Parks.

Dramatic declines in amphibian populations began to be reported worldwide in the late 1980s. Drost and Fellers [34] resurveyed a sampling transect within Yosemite National Park that had been previously surveyed for amphibians by Grinnell et al. in the early 20th Century (Yosemite National Park is located in the Sierra Nevada Range 60 km northwest of SEKI) and found dramatic declines in toads and frogs.

Surveys for amphibians and reptiles within SEKI might have increased in response to this news about amphibians, because the number of amphibian and reptile records peaked in the 1990s. Our analysis shows that the number of amphibian and reptile species decreased in SEKI from 1980 to the present at elevations below 2,500 m, while increasing at elevations above 3,000 m (Figure 5). No observations of foothill yellow-legged frogs (*Rana boylii Baird, 1854*) recorded since 1970, coupled with many observations before 1970, supports the personal observation by Daniel Boiano (May 20, 2014) that foothill yellow-legged frog has been extirpated from the Parks. The increase in amphibians and reptiles above 2,500 m might reflect the warming that has occurred at higher elevations, allowing additional amphibian and reptile species to live there.

Moritz et al. [35] resurveyed Grinnell's early-20th century small mammal surveys along an elevation transect that extending from 60 m to 3,300 m in elevation. After controlling for variation in species detectability, the authors found that (1) mammal species richness had decreased within all life zones, (2) species had moved up in elevation (the elevational ranges for half of the 28 species resurveyed had increased by an average of 500 m and formerly low-elevation species had expanded their ranges upward), and (3) the spatial ranges of high-elevation species had contracted. Moritz et al. determined that these results were consistent with a 3.7°C increase in minimum temperatures over the past 100 years.

Morelli et al. [36] resurveyed 47 sites in California where montane Belding's ground squirrels (*Urocitellus beldingi* Merriam, 1888) were recorded from 1902–1966. They found that *U. beldingi* had been extirpated from 42% of the sites where it had previously been found. There was no evidence that previously unoccupied new sites had become colonized. They also modeled changes in precipitation and temperature since 1900 and their model predicted adverse changes in ground squirrels at sites where *U. beldingi* had once lived but has since been extirpated. Morelli et al. attributed these extirpations of *U. beldingi* to a warmed climate that had resulted in a reduction of protective snow cover during the winter. The grizzly bear (*Ursus arctos* Linnaeus, 1758), and probably the wolverine (*Gulo gulo* Linnaeus, 1758), have also been extirpated from SEKI. Our study results add to those of Moritz et al. and Morelli et al., suggesting that mammal species richness has decreased since the 1980s in the Sierra Nevada Range (Figure 5).

The clumped distribution of plant data from SEKI through time, with most of the data recorded after 1985, made it impossible for us to do a temporal analysis of plant species richness since 1980. The evidence above for declines in vertebrate biodiversity in SEKI and elsewhere in the Sierra Nevada Range since the 1980s suggests that plant and invertebrate species richness might have decreased in SEKI since 1980. Thorne et al. [37] found that changes in vegetation composition in the central Sierra Nevada Range (located north of our study area) has been high in montane hardwood, Douglas-fir, and annual grassland habitat types; with greatest changes in foothill pine, ponderosa Pine, and blue oak-foothill pine habitat types. Most of these affected habitat types are found at relatively low elevations. The habitat types with the greatest changes are located at the lowest elevations where conditions were already warm to hot even before global warming. Thorne et al. concluded that potential causes for these changes include human disturbance, plant community succession, and climate change.

Considering invertebrates, Forister et al. [38] resurveyed sites in the Sierra Nevada previously surveyed for butterflies and found that species richness for butterflies in the Sierra Nevada has declined by 50% at the sites they revisited. They found that these declines were greatest at low elevations, where habitat destruction and the effects of global warming may have been the greatest.

The above evidence suggests the need for further studies to determine the status of all species, and in particular listed species, within SEKI. Furthermore, evidence for declines in species richness within protected national parks suggests the need to see how much species richness values have declined in areas outside of protected national parks and the factors involved in those declines.

The retired chief scientist from SEKI and the architect behind the SEKI WOD, Dave Graber, cautioned against over extrapolating from the SEKI WOD data. He noted that many scientists were active in the Parks from 1983–1991 installing Natural Resource Inventory Plots and adding a lot of new data to the WOD. Dave felt this large number of records might have skewed the WOD data, increasing the number of species observed in the 1980s relative to later decades (personal conversation on May 2, 2014). Also, as noted below, inputs into the WOD dropped off in 2003 when Graber was no longer as actively involved. Our use of rarefaction hopefully reduced or eliminated these temporal biases.

### How different is the wildlife observation data from SEKI relative to other parks?

Many national parks have wildlife observation databases but not all are as well maintained as those at SEKI. Rachel Moser, wildlife biologist at SEKI from 2000–2008, has worked in ten national parks. Moser believed the WOD at SEKI was much more extensive than the other ten national parks where she has worked. She credited this to the efforts made by Dave Graber to promote the WOD, as well as to the large number of scientists (National Park Service and U. S. Geological Society science staff) working at SEKI that contributed to the WOD (personal conversation on May 8, 2014).

When Dave Graber came to SEKI in 1980 he was surprised to learn that the National Park Service did not know what wildlife species lived in, had been lost to, or had been introduced to the Parks. During the early 1980s, Graber accumulated 1,000 records of wildlife observations from park naturalists. Graber later went on to encourage backcountry rangers to keep records of what wildlife they saw during their patrols. Graber estimated that backcountry rangers and the SEKI staff as a whole accounted for 50% and 90% of the records within the WOD, respectively. Dave Graber's job responsibilities were changed in 2003. As a consequence, the number of records going into the WOD dropped significantly after 2003 (Dave Graber, personal conversation on May 2, 2014).

### Applicability of this methodology to other natural areas

Each of the three data sets used in our analysis (park-wide systematic surveys, localized targeted studies of particular taxa, and chance wildlife observations) has both strengths and weaknesses for use in estimating the spatial and temporal distribution of species richness within SEKI.

Preserve-wide systematic surveys provide perhaps the highest quality data as they are often specific to a specific taxonomic group, or groups, and are conducted by experts. However, such surveys are expensive, often last only a year or two, may not find rare or transient species, and remain largely uncompleted. Thus, accuracy and spatial coverage are moderate to high for park-wide systematic surveys, but temporal and taxonomic inclusiveness may be low.

Localized data from targeted studies were often conducted by trained park staff or taxonomic experts for specific and varied purposes. Accuracy may be high but spatial coverage, temporal inclusiveness, and taxonomic inclusiveness may be low.

Chance wildlife observational data sets can contribute an abundance of data cheaply and are unique in that they can contribute continuous recordings of wildlife species throughout the year and for many years. These data sets may be the only information we have on some taxonomic groups. As a consequence, chance wildlife observation databases allow for moderately high spatial coverage of national parks if backcountry staff is utilized. However, observational data can lack the rigor of sampling by experts and can have biases in the types of species recorded (large, diurnal, easily identified, and rare species get the most attention). Recognizing that most observations within the Parks are not reported, and that some species may be misidentified helps researchers constrain conclusions based on these kinds of data. Temporal resolution may be high, spatial coverage moderately high, but accuracy and taxonomic inclusiveness may be low to medium. Vetting by subject experts is a necessity

Systematic surveys require skilled expertise and are expensive. Collection of chance wildlife observations by staff and visitors or local experts offers one relatively inexpensive way to gather some of the same information. Though the SEKI WOD represents an exemplary case, given the priority given to the WOD at SEKI, wildlife and plant lists can be economically produced for other sites. These efforts, however, require a strong effort to encourage observations from staff, scientists, and the interested public; require careful scrutiny of submitted observations - noting details, locations and the skill level of contributors; and require considerable investment in database management.

## Conclusions

As of May 2013, Natural Resources Condition Assessments have been completed for 70 national parks, are ongoing in 90 parks, and are planned for 110 additional national parks. Resource managers are asked to monitor biodiversity (species richness) within their jurisdiction, yet this monitoring can be prohibitively expensive. We estimated the spatial distribution of species richness for four taxonomic groups (birds, mammals, herpetofauna, and plants) within SEKI using only studies previously undertaken within the Parks and the Parks' long-term wildlife observation database. We are hopeful that our demonstration of the patterns that can be described by incidental wildlife observation databases can assist protected natural areas in their responsibilities to monitor biodiversity. Species richness for all four taxonomic groups varied with elevation, either peaking at middle elevations and then declining (mammals, plants, and total species richness) or declining consistently from low elevations to high elevations (herpetofauna and birds) (Figure 4). These observations are in line with general patterns described in the literature. Plants reached maximum species richness values at much higher elevations than did vertebrate taxa. Non-flying mammals reached maximum species richness values at higher elevations than did birds. Alpine and sagebrush plant communities had higher species richness values than did subalpine trees located just below them in elevation.

All three vertebrate taxa displayed declines in species richness since 1980 at low and middle elevations within SEKI. Mammal species richness declined at high elevations as well (Figure 5). Our analysis suggests that birds, amphibians, and reptiles species richness increased at high elevations, perhaps in response to climate warming. These conclusions are also in line with other published reports of declines in native species richness in the Sierra Nevada Range in recent years.

This information serves as valuable baseline information for the Parks as SEKI continues to monitor changes in species richness. Although the monitoring resources available at national parks like SEKI are large compared to other protected areas, we feel that the timely collection, vetting, and archiving of plant and wildlife observations, utilizing both staff and citizen scientists, can provide valuable monitoring of species richness at a manageably low cost.

## References

[1] Gamfeldt L, Hillebrand H, Jonsson PR (2008) Multiple functions increase the importance of biodiversity for overall ecosystem functioning. Ecology 89:1223–1231. 18543617 10.1890/06-2091.1

[2] National Park Service (2013) A natural resource condition assessment for Sequoia and Kings Canyon National Parks. Natural resource report NPS/SEKI/NRR—2013/665. Fort Collins, Colorado: National Park Service. 327p.

[3] Schwartz MW, Thorne J, Holguin A (2013) Appendix 20a – biodiversity. In:. Winford EM, Sydoriak C, Nydick K, Battles J, editors. A natural resource condition assessment for Sequoia and Kings Canyon National Parks. Natural resource report NPS/SEKI/NRR—2013/665. Fort Collins, Colorado: National Park Service. 80p.

[4] Gotelli NJ, Colwell RK (2001) Quantifying biodiversity: procedures and pitfalls in the measurement and comparison of species richness. Ecol Lett 4:379–391.

[5] Colwell RK, Mao CX, Chang J (2004) Interpolating, extrapolating, and comparing incidence-based species accumulation curves. Ecology 85:2717–2727.

[6] Chao A, Colwell RK, Lin C-W, Gotelli NJ (2009) Sufficient sampling for asymptotic minimum species richness estimators. Ecology 90:1125–1133. 19449706 10.1890/07-2147.1

[7] Gotelli NJ, Colwell RK (2011) Estimating species richness. In: Magurran AE and McGill BJ, editors. Biological diversity: frontiers in measurement and assessment. New York, New York: Oxford University Press. pp. 39–54.

[8] Siegel RB, Wilkerson RL (2005) Landbird inventory for Sequoia and Kings Canyon National Parks (2003–2004): final report. Point Reyes Station, CA: The Institute for Bird Populations. 237p.

[9] Thorne JH, Viers JH, Price J, Stoms DM (2009) Spatial patterns of endemic plants in California. Nat Area J 29:137–148.

[10] R Development Core Team (2008) R: A language and environment for statistical computing. Vienna, Austria: R Foundation for Statistical Computing.

[11] Oksanen J, Blanchet FG, Kindt R, Legendre P, Minchin PR, et al. (2011) Vegan: community ecology package. R package version 117-8. http://CRANR-projectorg/package=vegan.

[12] Hurlbert SH (1971) The nonconcept of species diversity: a critique and alternative parameters. Ecology 52:577–586. 28973811 10.2307/1934145

[13] Sanders NJ, Rahbek C (2012) The patterns and causes of elevational diversity gradients. Ecography 35:1–3.

[14] McCain CM, Grytnes JA (2010) Elevational gradients in species richness. In: Encyclopedia of Life Sciences (ELS). Chichester: John Wiley & Sons, Ltd. 1–10.

[15] Rahbek C (1995) The elevational gradient of species richness - a uniform pattern. Ecography 18:200–205.

[16] Rahbek C (2005) The role of spatial scale and the perception of large-scale species-richness patterns. Ecol Let 8:224–239.

[17] Grytnes JA, R. Vetaas O (2002) Species richness and altitude: a comparison between null models and interpolated plant species richness along the Himalayan Altitudinal Gradient, Nepal. Am Nat 159:294–304. 18707381 10.1086/338542

[18] Lomolino MV, Riddle BR, Whittaker RJ, Brown JH (2010) Biogeography. Sunderland, Mass.: Sinauer Associates. xiv, 878 p.

[19] Guo QF, Kelt DA, Sun ZY, Liu HX, Hu LJ, et al (2013) Global variation in elevational diversity patterns. Sci Rep-Uk 3. 10.1038/srep03007 PMC6505670 24157658

[20] Das AJ, Battles JJ, Stephenson NL, van Mantgem PJ (2007) The relationship between tree growth patterns and likelihood of mortality: a study of two tree species in the Sierra Nevada. Can J For Res 37:580–597.

[21] Tingley MW, Beissinger SR (2009) Detecting range shifts from historical species occurrences: new perspectives on old data. Trends Ecol. Evol. (Amst.) 24:625–633. 10.1016/j.tree.2009.05.009 19683829

[22] Tingley MW, Beissinger SR (2013) Cryptic loss of montane avian richness and high community turnover over 100 years. Ecology 94:598–609. 23687886 10.1890/12-0928.1

[23] Tingley MW, Koo MS, Moritz C, Rush AC, Beissinger SR (2012) The push and pull of climate change causes heterogeneous shifts in avian elevational ranges. Glob Chang Biol 18:3279–3290.

[24] Grinnell J, Storer TI (1924) Animal life in the Yosemite: an account of the mammals, birds, reptiles, and amphibians in a cross-section of the Sierra Nevada. Berkeley, Calif.: University of California Press. xviii, 752 p.

[25] Richerson PJ, Lum K (1980) Patterns of plant-species diversity in California - relation to weather and topography. Am Nat 116:504–536.

[26] Sánchez-González A, López-Mata L (2005) Plant species richness and diversity along an altitudinal gradient in the Sierra Nevada, Mexico. Divers Distrib 11:567–575.

[27] Lomolino MV (2001) Elevation gradients of species-density: historical and prospective views. Glob. Ecol Biogeogr 10:3–13.

[28] Grime J (1979) Plant strategies and vegetation process. John Wiley. pp. 222.

[29] Huston MA, Deangelis DL (1994) Competition and coexistence - the effects of resource transport and supply rates. Am Nat 144:954–977.

[30] Williams AE, Hendry K, Bradley DC, Waterfall R, Cragg-Hine D (2005) The importance of habitat heterogeneity to fish diversity and biomass. J Fish Biol 67:278–278.

[31] Hamilton AC, Perrott RA (1981) A study of altitudinal zonation in the montane forest belt of Mt. Elgon, Kenya/Uganda. Vegetatio 45:107–125.

[32] Austin J, Boiano D, Gammons D, Meyer E, Werner H (2013) Appendix 19 – native and non-native vertebrate species. In: N. P. Service, editors. A natural resource condition assessment for Sequoia and Kings Canyon National Parks Natural Resource Report NPS/SEKI/NRR. Fort Collins, Colorado: National Park Service.

[33] Grinnell J (1912) The Status of the California Condor in 1912. In: Hornaday W Teditor. Our vanishing wild life. New York: Charles Scribner's Sons. pp. 22–24. Drost CA and Fellers GM (1996) Collapse of a regional frog fauna in the Yosemite area of the California Sierra Nevada, USA. Biodivers Conserv 10: 414–425.

[34] Drost CA, Fellers GM (1996) Collapse of a regional frog fauna in the Yosemite area of the California Sierra Nevada, USA. Conservation Biology 10:414–425.

[35] Moritz C, Patton JL, Conroy CJ, Parra JL, White GC, et al (2008) Impact of a century of climate change on small-mammal communities in Yosemite National Park, USA. Science 322:261–264. 18845755 10.1126/science.1163428

[36] Morelli TL, Smith AB, Kastely CR, Mastroserio I, Moritz C, et al (2012) Anthropogenic refugia ameliorate the severe climate-related decline of a montane mammal along its trailing edge. Proc Biol Sci 279:4279–4286. 22896652 10.1098/rspb.2012.1301 PMC3441072

[37] Thorne JH, Morgan BJ, Kennedy JA (2008) Vegetation change over 60 Years in the Central Sierra Nevada. Madrono 55:223–237.

[38] Forister ML, McCall AC, Sanders NJ, Fordyce JA, Thorne JH, et al (2010) Compounded effects of climate change and habitat alteration shift patterns of butterfly diversity. Proc Natl Acad Sci U S A 107:2088–2092. 20133854 10.1073/pnas.0909686107 PMC2836664

